# The pregnane X receptor (PXR) and the nuclear receptor corepressor 2 (NCoR2) modulate cell growth in head and neck squamous cell carcinoma

**DOI:** 10.1371/journal.pone.0193242

**Published:** 2018-02-22

**Authors:** Juan Pablo Rigalli, Matthias Reichel, Tasmin Reuter, Guillermo Nicolás Tocchetti, Gerhard Dyckhoff, Christel Herold-Mende, Dirk Theile, Johanna Weiss

**Affiliations:** 1 Department of Clinical Pharmacology and Pharmacoepidemiology, University of Heidelberg, Heidelberg, Germany; 2 Institute of Experimental Physiology (IFISE-CONICET), Rosario, Argentina; 3 Molecular Cell Biology Group, Department of Otorhinolaryngology, Head and Neck Surgery, University of Heidelberg, Heidelberg, Germany; 4 Division of Neurosurgical Research, Department of Neurosurgery, University of Heidelberg, Heidelberg, Germany; Florida International University, UNITED STATES

## Abstract

Head and neck squamous cell carcinoma (HNSCC) is the sixth most frequent cancer worldwide. The pregnane X receptor (PXR) is a nuclear receptor regulating several target genes associated with cancer malignancy. We here demonstrated a significant effect of PXR on HNSCC cell growth, as evidenced in PXR knock-down experiments. PXR transcriptional activity is more importantly regulated by the presence of coactivators and corepressors than by PXR protein expression. To date, there is scarce information on the regulation of PXR in HNSCC and on its role in the pathogenesis of this disease. Coactivator and corepressor expression was screened through qRT-PCR in 8 HNSCC cell lines and correlated to PXR activity, determined by using a reporter gene assay. All cell lines considerably expressed all the cofactors assessed. PXR activity negatively correlated with nuclear receptor corepressor 2 (NCoR2) expression, indicating a major role of this corepressor in PXR modulation and suggesting its potential as a surrogate for PXR activity in HNSCC. To test the association of NCoR2 with the malignant phenotype, a subset of three cell lines was transfected with an over-expression plasmid for this corepressor. Subsequently, cell growth and chemoresistance assays were performed. To elucidate the mechanisms underlying NCoR2 effects on cell growth, caspase 3/7 activity and protein levels of cleaved caspase 3 and PARP were evaluated. In HNO97 cells, NCoR2 over-expression decreased cell growth, chemoresistance and increased cleaved caspase 3 levels, caspase activity and cleaved PARP levels. On the contrary, in HNO124 and HNO210 cells, NCoR2 over-expression increased cell growth, drug resistance and decreased cleaved caspase 3 levels, caspase activity and cleaved PARP levels. In conclusion, we demonstrated a role of PXR and NCoR2 in the modulation of cell growth in HNSCC. This may contribute to a better understanding of the highly variable HNSCC therapeutic response.

## 1. Introduction

Head and neck squamous cell carcinoma (HNSCC) comprises malignancies of the oral cavity, larynx, pharynx, nasal cavity and paranasal sinuses, representing the sixth most frequent cancer worldwide. HNSCC treatment consists of surgery, radiotherapy and chemotherapy. However, in spite of the improvement in the therapeutic strategies during the last decades, overall survival after 5 years remains between 40 and 50%. Molecular heterogeneity of HNSCC has been suggested as a reason underlying the variable response rate to the conventional therapeutic approaches [1].

The pregnane X receptor (PXR, NR1I2) is a nuclear receptor originally described as a master modulator of drug metabolism and disposition [2]. Moreover, recent investigations pointed out a role of PXR in cancer pathogenesis. For instance, Wang et al. described a PXR-driven increase in cell proliferation and metastatic potential in colon cancer [3]. In line with these observations, an induction of the anti-apoptotic genes bcl-2 and bcl-xL and a down-regulation of the pro-apoptotic genes BAK1 and p53 by PXR were reported [4,5]. Similar anti-apoptotic and pro-proliferative functions of PXR have been reported in models of breast-, endometrial-, ovarian- and prostate cancer [6]. On the contrary, an anti-proliferative and pro-apoptotic role of PXR was described in cervical cancer [7]. Although the expression of PXR in HNSCC is well-known [8], the impact of PXR on the malignant phenotype in HNSCC has not been investigated yet.

Mechanistically, PXR regulates the transcription of target genes through binding as a heterodimer with the retinoid X receptor alpha (RXRα) to response elements within the gene promoter [2]. Previously, we have characterized PXR expression levels and PXR intrinsic activity in a set of 8 HNSCC cell lines. Our data demonstrated no correlation between PXR protein expression and transcriptional activity, clearly indicating the presence of additional factors modulating PXR function [8]. Nuclear receptor cofactors are proteins physically interacting with nuclear receptors, thus affecting their functionality and biological effects. They can be divided into coactivators and corepressors with a higher expression of coactivators or corepressors resulting in an increased or reduced transcriptional activity of the associated receptor, respectively [9, 10]. PXR coactivators include the steroid receptor coactivators (SRCs) 1, 2 and 3, the peroxisome proliferator-activated receptor-gamma coactivator 1-alpha (PGC1α) and the p300 protein [11–13]. On the other side, PXR corepressors include the nuclear receptor corepressor 1 (NCoR1), the nuclear receptor corepressor 2 (NCoR2, also known as silencing mediator of retinoid and thyroid hormone receptor, SMRT) and the small heterodimer partner (SHP/NR0B2) [2, 10]. As previously described for PXR, a role of several cofactors in cancer pathogenesis has been already reported. For instance, higher SRC1, SRC2 or SRC3 expression was associated with a worse prognosis in breast cancer, prostate cancer or triple negative breast cancer patients, respectively [14–16]. In a similar way, corepressors can also influence cancer prognosis. For example, NCoR2 was described as a marker of earlier recurrence and poor outcome in breast carcinoma patients [17, 18]. To date, there is no information concerning PXR cofactor expression, their regulatory role on PXR transcriptional activity or their relevance for the malignant phenotype in HNSCC.

The aim of this work was to evaluate the effect of PXR and its regulatory cofactors on the cell growth, chemoresistance and apoptosis in HNSCC cell lines. A better understanding of the relevance of PXR and its regulation may help to identify potential prognostic markers better reflecting the molecular heterogeneity of HNSCC.

## 2. Materials and methods

### 2.1. Materials

Cisplatin, staurosporine and fetal bovine serum were from Sigma-Aldrich (Taufkirchen, Germany). Crystal violet, paclitaxel and 5-fluorouracil (5-FU) were from Applichem (Darmstadt, Germany). Primary antibodies used were: cleaved caspase 3 (Asp175, #9661, 1:1000) and cleaved poly ADP-ribose polymerase (PARP, Asp214, #9546, 1:2000) from Cell Signaling (Leiden, Netherlands); β-actin (AC-15, 1:10000) from Sigma-Aldrich (Taufkirchen, Germany); NCoR2 (ab24551, 1:1000) from Abcam (Cambridge, UK); GAPDH (G-9, 1:1000); Histone H1 (AE-4, 1:1000) and PXR (G-11, 1:100) from Santa Cruz Biotechnology (Heidelberg, Germany). Cleaved PARP, β-actin, GAPDH, Histone H1 and PXR antibodies were mouse monoclonal antibodies. Cleaved caspase 3 and NCoR2 were rabbit polyclonal antibodies. All antibodies were raised against the corresponding human antigens.

### 2.2. Cell lines

HNSCC cell lines (HNO97, HNO124, HNO150, HNO206, HNO210, HNO388, HNO413 and HNO432) used in the current work were established from intraoperatively obtained samples, characterized and cultured as already described (Table 1) [8, 19]. Cells were grown in polystyrene flasks and plates in Dulbecco’s modified Eagle’s medium supplemented with fetal bovine serum (10% v/v), penicillin (100 U/mL) and streptomycin (0.1 mg/mL). All cell lines were negatively tested for infection with *Mycoplasma* using the PCR Mycoplasma Test Kit (Applichem, Darmstadt, Germany) following the manufacturer’s instructions.

**Table 1:** HNSCC cell lines used in the current study.

Cell line	Localization	PXR intrinsic activity (arbitrary units)
HNO97	Oral cavity	35.9 ± 6.8
HNO124	Oral cavity	8.6 ± 0.7
HNO150	Larynx	2.6 ± 0.5
HNO206	Oropharynx	0.8 ± 0.4
HNO210	Larynx	16.7 ± 4.0
HNO388	Nasal cavity	2.8 ± 0.3
HNO413	Oropharynx	2.2 ± 0.5
HNO432	Hypopharynx	1.1 ± 0.2

Displayed is the localization of each HNSCC cell line used in the current study as well as the PXR intrinsic activity [8, 19].

### 2.3. PXR knock-down

To elucidate whether PXR modulates cell growth in HNSCC, PXR expression was silenced in a set of three cell lines basally displaying high PXR activity (HNO97, HNO124 and HNO210 cells). Cells were transfected with 4 μg of the pLKO.1-PXR shRNA vector (Sigma-Aldrich, Taufkirchen, Germany) encoding a short hairpin RNA (shRNA) targeting human PXR (PXR^-^ cells). Control cells were transfected with the pLKO.1-scrambled vector encoding a non-silencing shRNA (PXR^+^ cells) (Sigma-Aldrich, Taufkirchen, Germany). Transfections were performed by electroporation using the V-kit from Lonza (Basel, Switzerland) as previously described [8]. The efficacy of PXR knock-down was quantitatively verified at 72 h post-transfection through qRT-PCR (see section 2.4). PXR silencing was further verified at the protein level through western blot as previously described [8]. For cell growth evaluation, transfected cells were seeded in 96-well plates, incubated for 72 h and subjected to crystal violet staining as described in 2.5.

### 2.4. mRNA expression analysis

The expression of PXR, its heterodimerization partner RXRα, the coactivators SRC1, SRC2, SRC3, p300 and PGC1α as well as the corepressors NCoR1, NCoR2 and SHP was quantified at the mRNA level through qRT-PCR with calibrator-normalized relative quantification with efficiency correction using a LightCycler^®^ 480 device (Roche Applied Science, Mannheim, Germany). For this purpose, total RNA was isolated using the GenElute Mammalian Total RNA Miniprep Kit (Sigma-Aldrich, Taufkirchen, Germany) and reverse transcribed using the RevertAid H Minus First Strand cDNA Synthesis Kit (Thermo Fisher Scientific, Waltham, USA). qPCR reactions were performed using the ABsolute qPCR SYBR Green mix (Thermo Fisher Scientific, Waltham, USA). Specificity of the amplification was verified by conducting a melting curve with continuous fluorescence measurement. The most suitable housekeeping gene was selected among a panel consisting of 7 genes [*60S acidic ribosomal protein P0 (HUPO)*, *β2-microglobuline (B2MG)*, *beta-glucuronidase (BGU)*, *glucose-6-phosphate dehydrogenase (G6PDH)*, *hypoxanthine-guanine phosphoribosyltransferase (HPRT)*, *RNA polymerase II (RPII) and ribosomal protein L13 (RPL13)]*, using the geNorm software (Version 3.4, Center for Medical Genetics, Ghent, Belgium) [20]. *RPII* showed the highest stability in the different cell lines and thus was selected as a reference gene for the following expression studies. Primer sequences and thermal profiles are provided as supplementary material (S1 Table).

### 2.5. Cell growth

Cell growth was evaluated by using the crystal violet method as previously described [21]. Briefly, HNO97, HNO124 and HNO210 cells were transfected or transfected and treated, according to each particular experiment. After the corresponding incubation time, cells were stained with a crystal violet solution for 15 min at room temperature. Subsequently, cells were rinsed, crystal violet was solubilized in methanol and absorbance was measured at 555 nm.

### 2.6 Correlation between coactivator- and corepressor expression and PXR activity

PXR activity was quantified using a reporter system consisting of the plasmids pGL4.21-PXRRE and pGL4.74. pGL4.21-PXRRE codifies firefly luciferase under the control of two PXR response elements. The size and position of the inserts was corroborated by agarose gel electrophoresis and plasmid sequentiation [8]. pGL4.74 (Promega, Mannheim, Germany) codifies *Renilla* luciferase under the control of a constitutive promoter and was used for normalization purposes. PXR activity resulted from the ratio between firefly luciferase and *Renilla* luciferase signals, measured using the Dual-Glo® luciferase assay system (Promega, Mannheim, Germany). Correlation between PXR activity and coactivator- and corepressor expression was analyzed using GraphPad Prism 7.02 (GraphPad Software, San Diego, USA). Normal distribution of data was evaluated using the Kolmogorov-Smirnov test. Since datasets were not normally distributed, the correlation between expression and activity values was assessed using the non-parametric Spearman’s method.

### 2.7. NCoR2 over-expression and effect on PXR activity

PXR regulation by NCoR2 was further confirmed in a gain-of-function model. For this purpose, NCoR2 was over-expressed in HNO97, HNO124 and HNO210 cells, which originally display low basal NCoR2 expression and high PXR activity (Table 1) [8]. Cells were transfected with 1 μg of the pCMV6-NCoR2 plasmid (Clone RC212113, Origene, Rockville, USA) encoding the transcript variant 1 of the corepressor, previously described as the main NCoR2 isoform interacting with PXR [22]. Control cells were transfected with 1 μg of the empty pCMV6 vector (Clone PS100001, Origene, Rockville, USA). All transfections were performed as described [8]. NCoR2 over-expression was verified at the mRNA level at 12 h post-transfection as described in 2.4. NCoR2 expression at the protein level was evaluated through western blot as described previously [8, 21]. NCoR2 effect on PXR activity was assessed by cotransfection of the pCMV6-NCoR2 plasmid together with the PXR reporter system described in 2.6.

### 2.8. Effect of NCoR2 on cell growth and chemoresistance

The effect of NCoR2 on cell growth was evaluated by using the crystal violet method as previously described (see section 2.5). Briefly, HNO97, HNO124 and HNO210 cells were transfected with pCMV6-NCoR2 or with the pCMV6 empty plasmid as described in 2.7. Following, cells were seeded in 96-well plates and incubated for 72 h. Then, cells were stained and crystal violet absorbance was measured at 555 nm. To evaluate whether NCoR2 over-expression modulates resistance to chemotherapeutic agents usually administered in HNSCC treatment, transfected cells were seeded in 96-well plates (10000 cells/well for HNO97 and HNO124 cells and 5000 cells/well for HNO210 cells), incubated for 24 h and exposed to different concentrations of 5-FU (0.5–25000 μM), cisplatin (0.01–200 μM) or paclitaxel (0.01–500 nM) for 48 h. Then, crystal violet absorbance was measured. Cell viability was plotted against the decimal logarithm of drug concentration and fitted to a sigmoidal curve using Graph Pad Prism 7.02 (GraphPad Software, San Diego, USA). Resistance to the different chemotherapeutic agents was expressed in terms of the concentration leading to 50% of cell death (IC_50_).

### 2.9. Effect of NCoR2 on apoptosis

To evaluate whether apoptosis could be a mechanism underlying the NCoR2 effects on cell growth, caspase 3- and 7- activities were quantified using the Caspase-Glo® 3/7 Assay (Promega, Mannheim, Germany) following manufacturer’s instructions. Briefly, cells were transfected with NCoR2 over-expression- or the corresponding empty vector as described in 2.7, seeded in 96-well plates (12500 cells/well) and cultured for 24 h. Then, cells were incubated for 30 min with a luminogenic caspase 3/7 substrate. Subsequently, luminescence was measured using a GloMax luminometer (Promega, Mannheim, Germany). Caspase associated luminescence was normalized to the total number of cells, assessed using the CytoTox-Glo^TM^ assay (Promega, Mannheim, Germany) following manufacturer’s instructions. Staurosporine (1 μM, 24 h) was used as a positive control of apoptosis induction [23]. Furthermore, the protein levels of cleaved PARP (Asp214), which results from caspase 3-catalyzed proteolysis of PARP [24] as well as the protein levels of cleaved (active) caspase 3 (Asp175) were evaluated in pCMV6-NCoR2- and empty vector-transfected cells. For this purpose, total lysates were prepared with RIPA buffer 24 h post-transfection and subjected to SDS-PAGE and western blot as previously described [8, 21].

### 2.10. Statistical analysis

Statistical analysis was performed using GraphPad Prism 7.02 software (GraphPad Software, San Diego, USA). All experiments were repeated at least three times. Statistical differences between groups were evaluated using the Student’s t-test and significance was set at p<0.05. Data are presented as mean ± S.D.

## 3. Results

### 3.1. Effect of PXR on HNSCC cell growth

**Fig 1:**
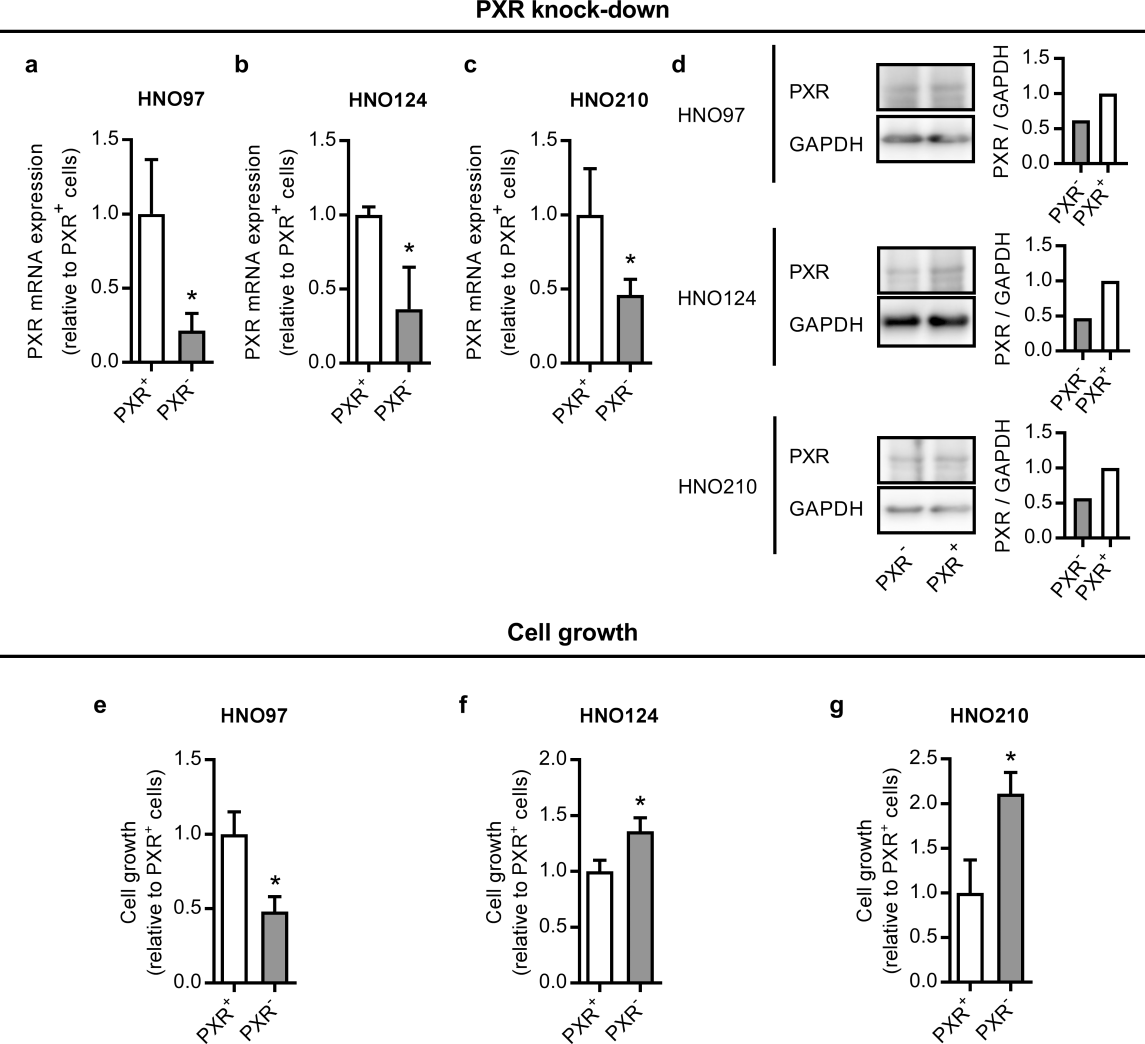
PXR knock-down and effect on cell growth in HNSCC cell lines. Cells were transfected with pLKO.1-PXR shRNA plasmid encoding a shRNA directed against human PXR (PXR^-^ cells) or the pLKO.1-scr vector encoding a scrambled, non-silencing shRNA (PXR^+^ cells). PXR mRNA expression was quantified through qRT-PCR in HNO97 **(a)**, HNO124 **(b)** and HNO210 **(c)** cells. *RPII* was used as a housekeeping gene. mRNA data were normalized to PXR expression in PXR^+^ cells, set to 1. Data are expressed as mean ± S.D. *significantly different from PXR^+^ cells, p<0.05, n = 3. PXR knock-down was also verified at the protein level through western blot analysis using GAPDH as a loading control. Representative images and histograms are shown for each cell line **(d)**. The effect of PXR knock-down on cell growth was assessed at 72 h post-transfection in HNO97 **(e)**, HNO124 **(f)** and HNO210 **(g)** cells using the crystal violet method. Results were normalized to cell growth in cells transfected with a non-silencing shRNA (PXR^+^ cells), set to 1. Data are expressed as mean ± S.D. *significantly different from PXR^+^ cells, p<0.05, n = 3.

To evaluate the modulation of cell growth by PXR, the expression of the nuclear receptor was silenced in HNO97, HNO124 and HNO210 cells using a specific shRNA. PXR expression at the mRNA level was down-regulated by 79%, 64% and 54% in HNO97 (Fig 1A), HNO124 (Fig 1B) and HNO210 PXR^-^ cells (Fig 1C), respectively. Silencing was further confirmed at the protein level (Fig 1D). Under these conditions, HNO97 PXR^-^ cells exhibited a decreased cell growth (-52%) compared to scrambled shRNA-transfected PXR^+^ cells (Fig 1E). On the contrary, HNO124 and HNO210 PXR^-^ cells exhibited an increase in the cell growth (+36% and +111%, respectively) (Fig 1F and 1G). These results confirm a modulation of cell growth by PXR in HNSCC, as previously described for other tumor types.

### 3.2. Expression of coactivators and corepressors in HNSCC cell lines

**Fig 2:**
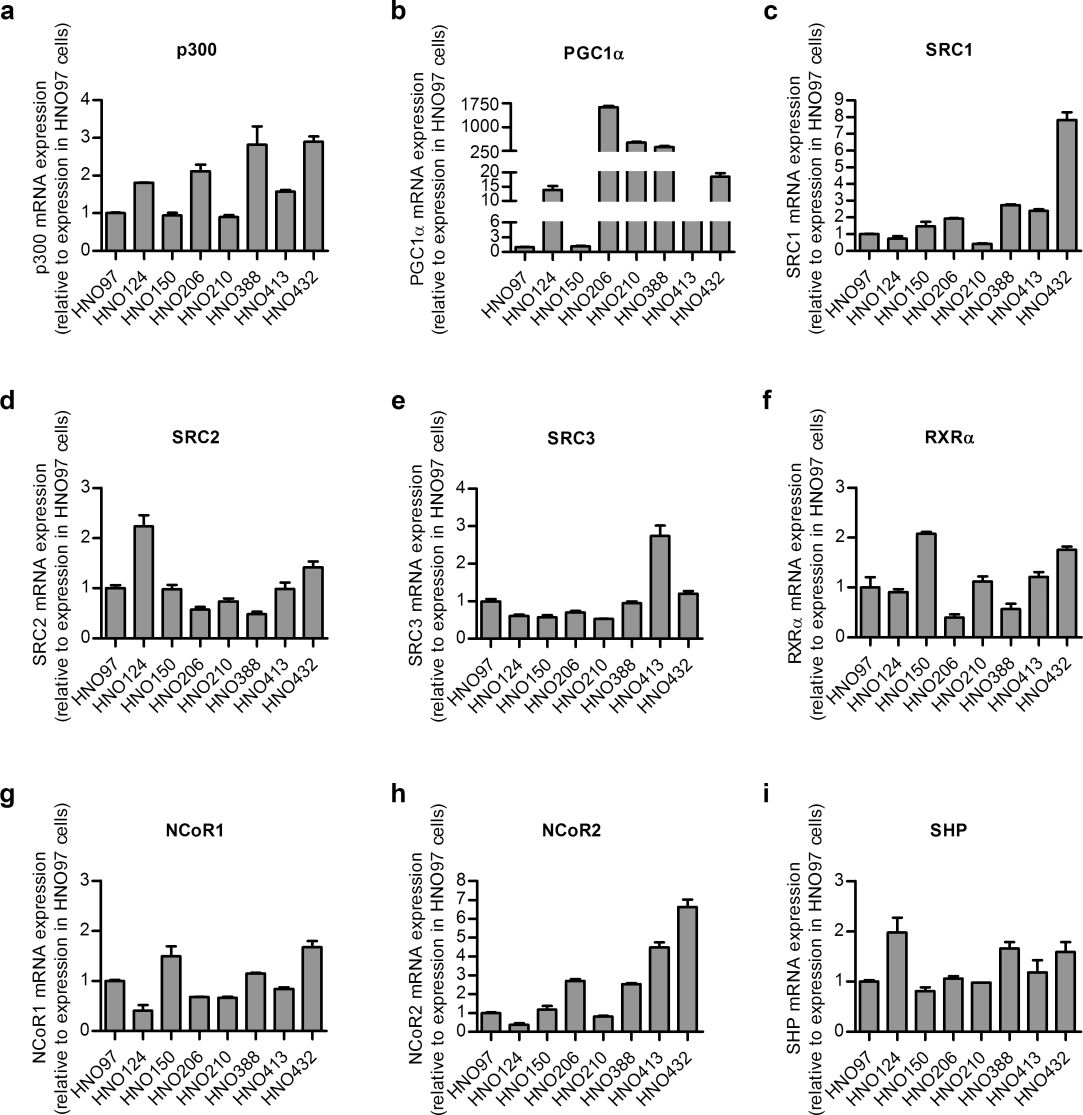
Cofactor expression in HNSCC cell lines. The expression of p300 **(a)**, PGC1α **(b)**, SRC1 **(c)**, SRC2 **(d)** and SRC3 **(e)** as nuclear receptor coactivators, RXRα **(f)** as PXR heterodimerization partner and NCoR1 **(g)**, NCoR2 **(h)** and SHP **(i)** as nuclear receptor corepressors was assessed at mRNA level in a set of 8 HNSCC cell lines through qRT-PCR. The expression of the different target genes was normalized to *RNA Polymerase II (RPII)* expression, used as housekeeping gene. Data are expressed as mean ± S.D. (n = 3). To allow for comparisons between cell lines, results were normalized to the expression in HNO97 cells, set to 1.

Considering the potential importance of PXR for HNSCC malignant phenotype and the well-known dissociation between PXR expression and PXR transcriptional activity in HNSCC [8], the expression pattern of PXR cofactors was assessed as a possible mechanism contributing to this observation. All the HNSCC cell lines evaluated showed a considerable mRNA expression of the coactivators p300, SRC1, SRC2, SRC3 (Fig 2A, 2C–2E), as well as of the corepressors NCoR1, NCoR2 and SHP (Fig 2G–2I). In addition, the PXR partner RXRα also exhibited a detectable signal in all the cell lines (Fig 2F). PGC1α expression was detected in all cell lines, whereas HNO206, HNO210 and HNO388 cells exhibited notably higher expression levels than the other cell lines evaluated (Fig 2B). These data confirm the presence of the above-mentioned cofactors in HNSCC, as demonstrated for other tumor diseases, and suggest a potential role in the modulation of PXR in this tumor disease.

### 3.3. Correlation between coactivator- and corepressor expression and PXR activity

The association between the expression of each coactivator and corepressor (Fig 2) and PXR activity data previously obtained by our group (Table 1) [8] was assessed in the panel of 8 HNSCC cell lines through the Spearman’s correlation method. Results for all cofactors are presented in Table 2. A significant negative correlation was observed between NCoR2 expression and PXR activity, indicating indeed a major role of this corepressor in the modulation of PXR activity in HNSCC (Table 2). In contrast, expression of p300, PGC1α, SRC1, SRC2, SRC3, NCoR1, SHP and RXRα exhibited no significant correlation with PXR activity (Table 2).

**Table 2:** Correlation between coactivator- and corepressor expression and PXR transcriptional activity.

Coactivator or corepressor	r	p-value
p300	-0.571	0.15
PGC1α	-0.381	0.36
SRC1	-0.690	0.07
SRC2	0.167	0.70
SRC3	-0.333	0.43
RXRα	-0.048	0.94
NCoR1	-0.310	0.46
**NCoR2**	**-0.810**	**0.02**
SHP	-0.143	0.75

Expression of coactivators and corepressors as well as of the PXR partner RXRα was correlated to the PXR activity in the set of 8 HNSCC cell lines used in the present study. Displayed are the Spearman’s correlation coefficients (r) and the p-value, n = 3. Statistical significance was set at p<0.05.

### 3.4. Effect of NCoR2 over-expression on PXR activity

**Fig 3:**
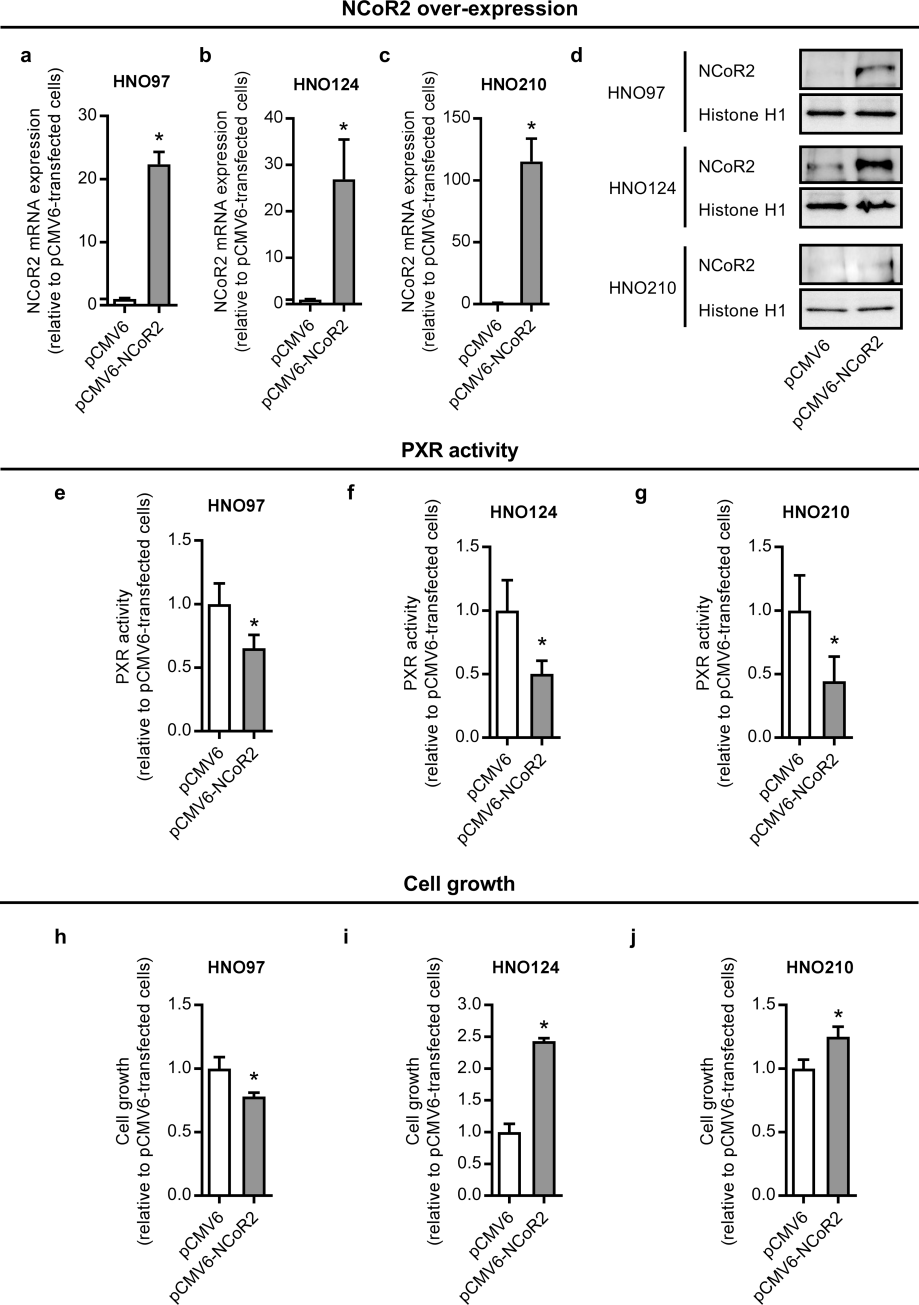
Effect of NCoR2 over-expression on PXR activity and cell growth in HNO97, HNO124 and HNO210 cells. Cells were transfected with the pCMV6-NCoR2 plasmid (or the empty vector pCMV6). NCoR2 over-expression was quantified at the mRNA level through qRT-PCR at 12 h post-transfection in HNO97 **(a)**, HNO124 **(b)** and HNO210 **(c)** cells. *RPII* was used as housekeeping gene. mRNA data were normalized to NCoR2 expression in cells transfected with the pCMV6 empty vector, set to 1. NCoR2 over-expression was verified at the protein level at the same time point through western blot analysis using histone H1 as a loading control. Representative images are shown for each cell line **(d)**. PXR activity was measured at 48 h post-transfection in HNO97 **(e)**, HNO124 **(f)** and HNO210 **(g)** cells. Cell growth was measured at 72 h post-transfection in HNO97 **(h)**, HNO124 **(i)** and HNO210 **(j)** cells using the crystal violet method. Results were normalized to PXR activity or cell growth in cells transfected with the pCMV6 empty vector, set to 1. All data are expressed as mean ± S.D. *significantly different from pCMV6-transfected cells, p<0.05, n = 3–4.

To confirm NCoR2 role as a negative modulator of PXR activity, the nuclear receptor was over-expressed in three selected cell lines. Transfection of HNO97, HNO124 and HNO210 cells with the pCMV6-NCoR2 plasmid resulted in a significant increase in NCoR2 mRNA expression at 12 h post-transfection (+2136%, +2592% and +11408% in HNO97, HNO124 and HNO210 cells, respectively) compared to empty vector transfected cells (Fig 3A–3C). Over-expression was also verified at the protein level (Fig 3D). As expected, NCoR2 over-expression resulted in a significant decrease in PXR activity (-35%, -50% and -56% in HNO97, HNO124 and HNO210 cells, respectively) (Fig 3E–3G), further confirming a major role of NCoR2 in the regulation of this nuclear receptor in HNSCC.

### 3.5. Effect of NCoR2 over-expression on cell growth and chemoresistance

PXR and NCoR2 have been reported to modulate malignancy of cancer types other than HNSCC. We here observed a cell line-specific effect of NCoR2 over-expression with a decrease in HNO97 cell growth (-22%) (Fig 3H) and, on the contrary, an increase in HNO124 and HNO210 cell growth (+143 and +25%, respectively) compared to empty vector transfected cells (Fig 3I and 3J).

In addition, NCoR2 over-expression modified the resistance to chemotherapeutic agents usually administered in HNSCC treatment. In particular, NCoR2 over-expressing HNO97 cells exhibited a diminished resistance to cisplatin and paclitaxel, whereas NCoR2 over-expression resulted in an enhanced resistance to 5-FU, cisplatin and paclitaxel in HNO124 and HNO210 cells (Table 3).

**Table 3:** Drug resistance assays.

Cell line		5-FU (μM)	Cisplatin (μM)	Paclitaxel (nM)
HNO97	pCMV6	6.7 ± 2.1	6.6 ± 0.2	7.7 ± 0.6
	pCMV6-NCoR2	7.3 ± 1.7	4.3 ± 0.5*	5.4 ± 0.9*
HNO124	pCMV6	10.9 ± 0.7	10.2 ± 0.6	4.6 ± 0.1
	pCMV6-NCoR2	14.2 ± 1.6*	12.6 ± 0.9*	6.0 ± 0.5*
HNO210	pCMV6	5.5 ± 0.5	1.6 ± 0.2	2.0 ± 0.6
	pCMV6-NCoR2	8.0 ± 1.2*	2.3 ± 0.1*	3.3 ± 0.4*

Displayed are the IC_50_ of 5-FU, cisplatin and paclitaxel in pCMV6 and pCMV6-NCoR2 transfected HNO97, HNO124 and HNO210 cells.

*significantly different from the corresponding pCMV6-transfected cells, p<0.05, n = 3.

### 3.6. Effect of NCoR2 over-expression on apoptosis

**Fig 4:**
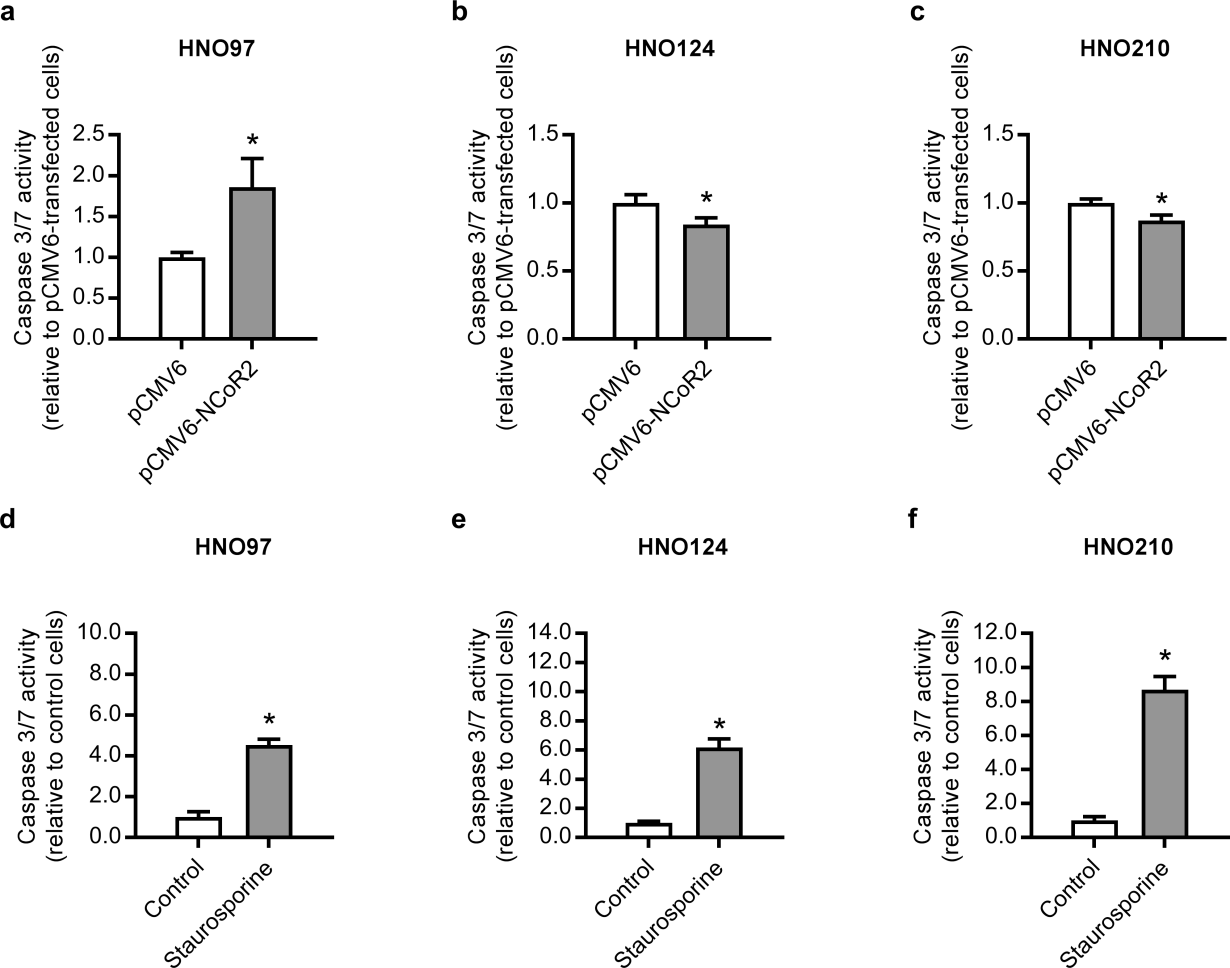
Caspase activity in NCoR2 over-expressing HNO97, HNO124 and HNO210 cells. Cells were transfected with pCMV6-NCoR2 plasmid (or the empty vector pCMV6). Caspase activity was assessed at 24 h post-transfection in HNO97 **(a)**, HNO124 **(b)** and HNO210 **(c)** cells. Results were normalized to the corresponding activity in cells transfected with the pCMV6 empty vector, set to 1. *significantly different from pCMV6-transfected cells, p<0.05, n = 3. As a positive control, cells were treated with staurosporine (1 μM, 24 h), a known inductor of apoptosis. Subsequently, caspase activity was assessed in HNO97 **(d)**, HNO124 **(e)** and HNO210 **(f)** cells. Results were normalized to the corresponding measurements in control cells (vehicle treated), set to 1. Data are expressed as mean ± S.D. *significantly different from control cells, p<0.05, n = 3.

**Fig 5:**
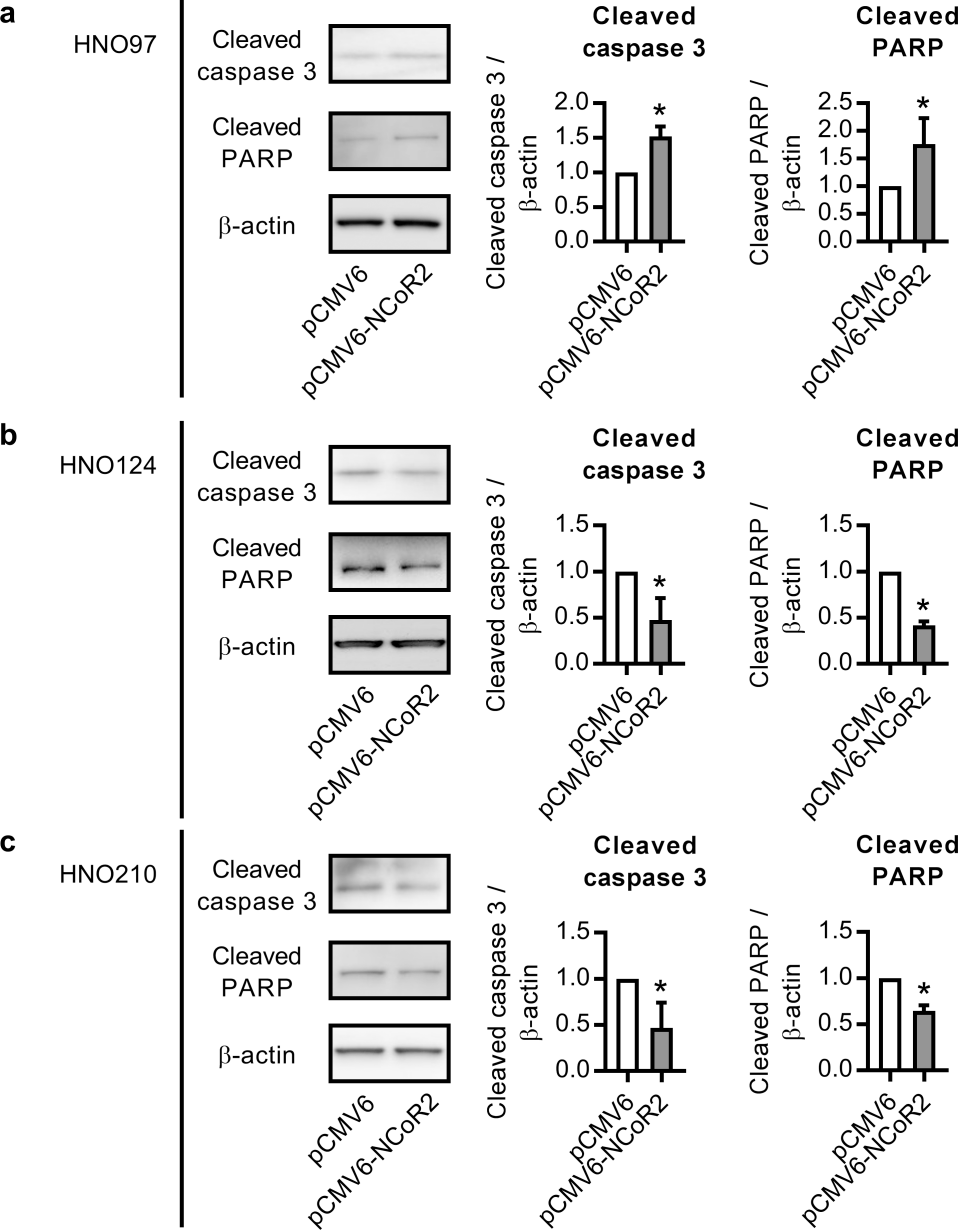
Cleaved caspase 3 and cleaved PARP expression in NCoR2 over-expressing HNO97, HNO124 and HNO210 cells. Cells were transfected with pCMV6-NCoR2 plasmid (or the empty vector pCMV6). Cleaved caspase 3 and cleaved PARP protein levels were evaluated at 24 h post-transfection in HNO97 **(a)**, HNO124 **(b)** and HNO210 **(c)** cells through western blot. β-actin was used as a loading control. Representative western blot detections are shown for each cell line. *significantly different from pCMV6-transfected cells, p<0.05, n = 3.

To evaluate whether changes in the apoptosis rate underlie the effect of NCoR2 over-expression on cell growth, we evaluated caspase 3/7 activities. HNO97 cells showed an increase in caspase 3/7 (+86%) activity by NCoR2 over-expression cells compared to empty vector transfected cells (Fig 4A), agreeing well with the reduced cell growth. On the contrary, NCoR2 over-expressing HNO124 and HNO210 cells exhibited a decrease in caspase 3/7 activity (-14% and -13%, respectively) (Fig 4B and 4C), indicating a reduction of the cell population undergoing apoptosis and probably explaining the increase in the cell growth previously observed (Fig 3). Treatment with staurosporine (1 μM, positive control for apoptosis) for 24 h resulted in a significant increase in caspase 3/7 activity in all the cell lines used (Fig 4D–4F). In addition, NCoR2 over-expression resulted in a significant increase in the expression of cleaved caspase 3 and cleaved-PARP in HNO97 cells (Fig 5A). On the contrary, HNO124 and HNO210 cells exhibited a significant decrease in cleaved caspase 3 and cleaved PARP (Fig 5B and 5C) agreeing well with the reduced caspase 3/7 activity and supporting an inhibitory effect of NCoR2 on the apoptosis in these cell lines.

## 4. Discussion

Molecular heterogeneity is likely to be one of the reasons underlying the variable response to different therapeutic strategies and the poor overall survival in HNSCC [1]. Nuclear receptors and their cofactors modulate several signal pathways related to cancer malignancy. In the present work, we described opposite effects of PXR on cell growth in HNSCC according to the particular cell line (Fig 1). Differential effects of PXR on cell growth, even within the same cancer type, have been described for instance in colon cancer. Here, an inhibition of p53 transcriptional activity and a promotion of the malignant phenotype have been described [25]. Similarly, stimulation of tumor malignancy by PXR via the activation of the fibroblast growth factor 19 (FGF19) was reported [3]. Conversely, p21 up-regulation and inhibition of proliferation by PXR have been also described in another model of the same disease [26]. Our results here, demonstrating a significant role of PXR in HNSCC pathogenesis, highlight the importance of further studying this nuclear receptor as a factor contributing to HNSCC molecular heterogeneity and variable therapeutic response.

Previously, we have described the dissociation between PXR expression and transcriptional activity in HNSCC. Indeed, the differential activation of a reporter gene under the control of a PXR responsive promoter could not be explained in terms of PXR protein levels [8]. Coactivators and corepressors are key determinants of PXR activity, especially in the absence of an external ligand [2, 10, 13]. We here characterized the expression pattern of the most important PXR coactivators and corepressors in a set of HNSCC cell lines. All coactivators and corepressors investigated were expressed in all the HNSCC cell lines used (Fig 2). So far, clinical evidence suggests a crucial role of these accessory proteins in cancer prognosis. For instance, several clinical studies associated p300 expression with melanoma, colorectal or lung cancer prognosis [27–29]. For SRC1, -2 and -3 a similar role was suggested in diverse malignancies [14–16]. Likewise, corepressors have also been associated with response to treatment and prognosis in lung, liver and breast cancer [17, 18]. In general, cancer initiation, promotion, progression, metastases formation as well as response to treatment are the result of the interplay of a large number of genes [30]. Among them, cyclooxygenase 2 (COX2), the chemokine receptor CXCR4 and the matrix metalloproteinase 9 (MMP9) as well as apoptosis related genes are associated with the malignant phenotype of HNSCC and have been shown to be regulated by variable levels of nuclear receptor cofactors, albeit in other models [31–33]. Noteworthy, a modulation of COX2 and CXCR4 levels by NCoR2 was observed [34]. Our results demonstrating a significant expression of p300, SRC1, NCoR1 and NCoR2, among other cofactors, suggest a possible regulation of the above-mentioned malignancy associated genes also in HNSCC.

In the present work, we demonstrated a negative correlation (r = -0.810; p = 0.02) between NCoR2 expression and PXR activity (Table 2). Furthermore, this regulation was confirmed using a gain-of-function model in a subset of 3 cell lines exhibiting lower basal NCoR2 expression and higher PXR activity. These observations clearly suggest NCoR2 expression as a surrogate for PXR activity in HNSCC. Taking into consideration the association between NCoR2, PXR and cancer malignancy, we evaluated whether NCoR2 can indeed alter cell growth in HNSCC. Interestingly, our results demonstrated a cell line-specific effect displaying the same pattern exhibited in PXR knock-down experiments. NCoR2 over-expression (i.e. lower PXR activity) inhibited cell growth in the HNO97 cell line, agreeing well with the inhibition in cell growth observed under PXR silencing (Fig 1). Conversely, NCoR2 over-expression stimulated growth in HNO124 and HNO210 cells (Fig 3), also agreeing with the increased cell growth in HNO124 and HNO210 PXR^-^ cells (Fig 1). This similarity between PXR silencing and NCoR2 over-expression experiments further indicates a functional association between PXR and NCoR2, as described in other models [2, 10]. In addition, NCoR2 over-expression led to an increase in caspase 3/7 activity in HNO97 cells (Fig 4) and an increase in the protein levels of cleaved caspase 3 and cleaved PARP (Fig 5), possibly accounting for the decrease in the cell growth in this cell line. On the contrary, NCoR2 led to a decrease of caspase activation in NCoR2 over-expressing HNO124 and HNO210 cells, as observed through activity measurements (Fig 4) and western blot studies (Fig 5), in accordance with the effects on cell growth. Noteworthy, our results agree with previous reports demonstrating a modulation of apoptosis by this same corepressor. For example, Song et al. described an increase in apoptosis of non-Hodgkin lymphoma cell lines by NCoR2 [35]. On the contrary, NCoR2 reduced apoptosis in breast cancer cell lines [23]. Our data as well as previous studies, although remarking the NCoR2 importance in cancer growth, evidence differential NCoR2 effects in different models. Noteworthy, HNO97 cells were derived from a tumor with a higher grading than HNO124 and HNO210 cells [19]. If these observations translated to an *in vivo* situation, NCoR2 expression might have opposite prognostic implications according to the molecular milieu, tumor grading and stage of the disease.

Chemotherapy represents one of the milestones of HNSCC treatment. In the present work, we observed an increased HNO97 cell sensitivity to cisplatin and paclitaxel through NCoR2 over-expression (Table 3), probably associated with the reduced cell growth and increased apoptosis exhibited. On the contrary, NCoR2 drove an increase in HNO124 and HNO210 cell resistance to 5-fluorouracil, cisplatin and paclitaxel, agreeing well with the enhanced cell growth and reduced apoptosis observed. Response to chemotherapeutic agents is governed by multiple factors. Among them, proteins like p53, COX2 and CXCR4 can also contribute to the enhanced chemoresistance, as already demonstrated in clinical studies [33, 36–38]. Since p53, COX2 and CXCR4 are well-known PXR and NCoR2 targets, they constitute potential mediators of NCoR2 effects on chemoresistance [34, 39]. If such association took place *in vivo*, the analysis of NCoR2 expression levels may contribute to a better prediction of chemotherapy response in HNSCC.

In conclusion, we have described a modulation of cell growth by PXR in HNSCC. Furthermore, we demonstrated the role of NCoR2 in the modulation of PXR activity in HNSCC, constituting this corepressor as an indicator of PXR functionality in HNSCC. In line with these findings, we demonstrated a major role of NCoR2 in the regulation of cell growth, chemoresistance and apoptosis, probably as a consequence of its interaction with PXR. Although these findings should be further validated in more complex experimental settings (e.g. animal models, clinical studies) and extrapolations from *in vitro* studies to an *in vivo* situation should always be done cautiously, these results suggest a role of NCoR2 in the malignant phenotype of HNSCC and point out a potential role of this protein as a biomarker.

## Supporting information

S1 Table Primer sequences and thermal profiles used in qPCR. * NCoR2 primers amplify only the transcript variant 1 of the corepressor. F, forward primer; R, reverse primer.
(DOCX)

S1 Dataset Data presented in figures and tables. (XLSX)
