# Supplementary material for: The pregnane X receptor (PXR) and the nuclear receptor corepressor 2 (NCoR2) modulate cell growth in head and neck squamous cell carcinoma
Source: PLoS One. 2018 Feb 22;13(2):e0193242. doi: 10.1371/journal.pone.0193242 (PMC5823449; doi:10.1371/journal.pone.0193242)
Supplement: S1 Table — * NCoR2 primers amplify only the transcript variant 1 of the corepressor. F, forward primer; R, reverse primer. (DOCX) [file pone.0193242.s001.docx]

| Gene | Primers | Thermal Profile |
| --- | --- | --- |
| *NCoR2** | F: 5´GGGTAAATATGACCAGTGGGAAGAG3´ | 15 min, 95 °C; 40 cycles: 15 s, 95 °C; 30 s, 60 °C; 30 s, 72 °C |
|  | R: 5´TGGCATTCAGAGGGTTAAAAGC3´ |  |
| *p300* | F: 5´GACCCTCAGCTTTTAGGAATCC3´ | 15 min, 95 °C; 40 cycles: 15 s, 95 °C; 30 s, 58 °C; 30 s, 72 °C |
|  | R: 5´TGCCGTAGCAACACAGTGTCT3´ |  |
| *PGC1α* | F: 5´CCCATTTGAGAACAAGACTAT3´ | 15 min, 95 °C; 40 cycles: 15 s, 95 °C; 30 s, 60 °C; 30 s, 72 °C |
|  | R: 5´GGTTATCTTGGTTGGCTTT3´ |  |
| *PXR* | F: 5´CCCAGCCTGCTCATAGGTTC3´ | 15 min, 95 °C; 45 cycles: 15 s, 95 °C; 30 s, 60 °C; 30 s, 72 °C |
|  | F: 5´CTGTGATGCCGAACAACTCC3´ |  |
| *RPII* | F: 5´GCACCACGTCCAATGACAT3´ | 15 min, 95 °C; 40 cycles: 15 s, 95 °C; 30 s, 61 °C; 30 s, 72 °C |
|  | R: 5´GTGCGGCTGCTTCCATAA3´ |  |
| *RXRα* | F: 5´TGGCAAGGACCGGAACGAGA3´ | 15 min, 95 °C; 40 cycles: 15 s, 95 °C; 30 s, 62 °C; 50 s, 72 °C |
|  | R: 5´GCGGCGCCTCCAGCATCTCCATA3´ |  |
| *SHP* | F: 5´TGGCTTCAATGCTGTCTGGAGT3´ | 15 min, 95 °C; 40 cycles: 15 s, 95 °C; 30 s, 60 °C; 30 s, 72 °C |
|  | R: 5´CTGGCACATCGGGGTTGAAGA3´ |  |
| *SRC1* | F: 5´AGGCCCAGAGCCAGTTTAC3´ | 15 min, 95 °C; 40 cycles: 15 s, 95 °C; 30 s, 60 °C; 30 s, 72 °C |
|  | R: 5´CAGGATCTCCGATTTGATGGTTA3´ |  |
| *SRC2* | F: 5´AGGCAACCTGTTCCCAAAC3´ | 15 min, 95 °C; 40 cycles: 15 s, 95 °C; 30 s, 56 °C; 30 s, 72 °C |
|  | R: 5´ACTGGCTTCAGCAGTGTCAG3´ |  |
| *SRC3* | F: 5´GTCATTCCTCCTTGACCAACTC3´ | 15 min, 95 °C; 40 cycles: 15 s, 95 °C; 30 s, 58 °C; 30 s, 72 °C |
|  | R: 5´ATCCCTGTCCAGCAGGTATCTA3´ |  |
